# Synthesis of Novel Ternary Dual Z-scheme AgBr/LaNiO_3_/g-C_3_N_4_ Composite with Boosted Visible-Light Photodegradation of Norfloxacin

**DOI:** 10.3390/molecules25163706

**Published:** 2020-08-14

**Authors:** Junjiao Zhang, Zhengru Zhu, Junchao Jiang, Hong Li

**Affiliations:** 1 School of Geography, Liaoning Normal University, Dalian 116029, China; zjjcaihong@outlook.com (J.Z.); zhengruzhu@lnnu.edu.cn (Z.Z.); 2 Department of Basic, Dalian Naval Academy, Dalian 116018, China; lswlx903@163.com

**Keywords:** g-C_3_N_4_, dual Z-scheme, ternary photocatalyst, norfloxacin, photocatalysis

## Abstract

Promoting the separation of photogenerated charges and enhanced optical absorption capacity is the main means to modify photocatalytic capacities to advance semiconductor photocatalyst applications. For the first time, a novel ternary photocatalyst for dual Z-scheme system AgBr/LaNiO_3_/g-C_3_N_4_ (ALG) was prepared via a modest ultrasound-assisted hydrothermal method. The results indicated that LaNiO_3_ nanoballs and AgBr nanoparticles were successfully grown on the surface of g-C_3_N_4_ nanosheets. A dual Z-scheme photocatalytic reaction system could be constructed based on the energy band matching within AgBr, LaNiO_3_ and g-C_3_N_4_. Metallic Ag during the photocatalytic reaction process acted as the active electrons transfer center to enhance the photocatalytic charge pairs separation. The chemical composition of ALG was optimized and composites with 3% AgBr, 30% LaNiO_3_ and 100% g-C_3_N_4_ which was noted as 3-ALG displayed the best photocatalytic performance. A total of 92% of norfloxacin (NOR) was photodegraded within two hours over ALG and the photodegradation rate remained >90% after six cycles. The main active species during the degradation course were photogenerated holes, superoxide radical anion and hydroxyl radical. A possible mechanism was proposed based on the synergetic effects within AgBr, LaNiO_3_ and g-C_3_N_4_. This work would offer a credible theoretical basis for the application of dual Z-scheme photocatalysts in environment restoration.

## 1. Introduction

In previous decades, antibiotics were widely used for the treatment and prevention of bacterial infections in human and animal healthcare. A large number of antibiotic wastewater is discharged into the environment without appreciating treatment [1]. Especially the fluoroquinolone antibiotic, norfloxacin (NOR), which was extensively used as a therapeutic and prophylactic antimicrobial agent in clinical medical treatment, aquaculture, and animal husbandry [2]. NOR has been detected in rivers, lakes and ground water [3]. The continuous accumulation of NOR can damage the security and stability of the natural environment.

Numerous researches had found that semiconductor photocatalytic technology can be efficacious means of solving the growing environmental pollution and energy crisis around the world [4,5,6,7]. However, common photocatalyst are limited in application for narrow light absorption range, poor stabilization, and lower photodegradation efficiency [8,9]. To improve the photocatalytic performance, abundant research was carried out concentrating on the formation of Z-scheme heterojunction [10,11], especially dual Z-scheme system photocatalyst [12]. In the course of a photocatalytic reaction over a dual Z-scheme photocatalyst, benefited from the nice matching of energy bands, photogenerated charge pairs are effectively separated, transferred. Finally the reaction center could be formed duo to the accumulation of conduction band (CB) and valence band (VB) to remove the organic pollution in water.

Nowadays, g-C_3_N_4_ has a great application prospect for removal of organic contamination from water [13]. It has the advantages of controllable, inexpensive synthesis, and stable chemical properties [14,15]. But wide band gap and high charge recombination were the shortcoming of g-C_3_N_4_, It still needs essential improvement. For the improvement of this situation and to make it more suitable for practical application, it needs to be modified and compounded with other activated materials, especially establishing the dual Z-scheme system photocatalyst according to the energy bands matching of related materials. 

Perovskite structure semiconductor possesses excellent performance in fuel cells, catalytic combustion, conductive materials, and photocatalysis [16,17]. Among them, a p-type semiconductor, LaNiO_3_, has promising applications in the field of photodegradation of organic contaminants and in hydrogen production benefitting from suitable band gap and electronic magnetic property. Many photocatalytic composites were successfully prepared, such as LaNiO_3_/ZnIn_2_S_4_ [18], LaNiO_3_/SnS_2_ [19], LaNiO_3_/CdS [20] and LaNiO_3_/TiO_2_ [21], which have made some progress in hydrogen production, dye degradation and tetracycline hydrochloride removal. Besides, as a semiconductor material with good photosensitivity, silver bromide (AgBr) is easy to synthesize, possesses stable physical and chemical properties, a narrow band gap, and outstanding renewability [22,23]. What’s more, numerous heterojunctions were synthesized based on AgBr according to previous researches such as AgPO_3_/AgBr/g-C_3_N_4_ [24], BiOBr/AgBr/LaPO_4_ [25], and AgBr/p-g-C_3_N_4_ [26]. Because AgBr possesses a narrow band gap, strong photosensitivity and high charge separation ability, also AgBr is easiest to produce metallic Ag under illumination, which can promote electron transfer in heterojunctions and improve charge separation efficiency. In addition, LaNiO_3_ and AgBr have good energy band matching with g-C_3_N_4_, which is an excellent choice for the formation of a dual Z-scheme system over the heterojunction to promote photocatalytic reactions. However, the research concerning on dual Z-scheme reaction system over AgBr/LaNiO_3_/g-C_3_N_4_ has not been reported until now to our knowledge.

Herein, AgBr/LaNiO_3_/g-C_3_N_4_ (ALG) ternary component dual Z-scheme photocatalyst was prepared by an ultrasound-assisted hydrothermal method. The samples obtained were further characterized by XRD, FT-IR, XPS, SEM, TEM, DRS, BET, PL, EIS. We also examined the photodegradation of norfloxacin (NOR) under xenon lamp in aqueous solution over ALG. Benefiting from the energy band matching and activated materials modified, this ternary photocatalyst has remarkable optical absorption capacity and excellent photocatalytic decomposition of NOR. The intentionally constructed dual Z-scheme not only improves the separation of charge pairs but also provides more active species for photodegradation. A probable reaction mechanism within the dual Z-scheme over the photocatalyst was proposed depended on the relative energy bands of the three materials.

## 2. Results and Discussion

### 2.1. Microstructure and Surface Morphology

**Figure 1:**
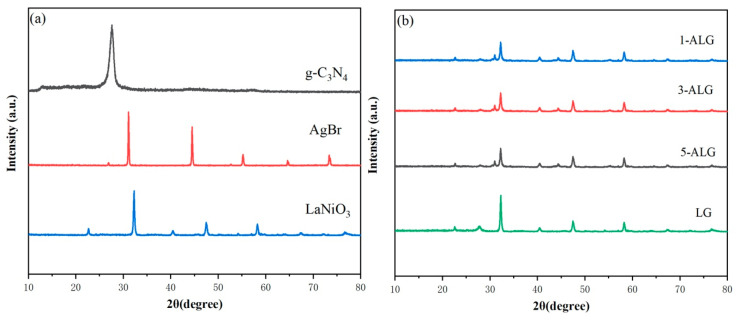
XRD patterns of bare g-C_3_N_4_, AgBr, LaNiO_3_ (**a**), 1-ALG, 3-ALG and 5-ALG (**b**).

The crystal structures of samples were characterized using XRD and the patterns were depicted in Figure 1. It was obvious that the diffraction peaks of AgBr, g-C_3_N_4_, and LaNiO_3_ as-prepared, were matched well with standard PDF card AgBr (JCPDS 64-38) [27], g-C_3_N_4_ (JCPDS 87-1526) [28], and LaNiO_3_ (JPCDS 33-0711) [29], respectively. To be specific, the characteristic peaks of AgBr found at 26.92°, 31.01°, 44.39°, 52.66°, 55.18°, 64.58°, and 73.32° corresponded to (111), (200), (220), (311), (222) and (400) crystal faces, the diffraction peaks of g-C_3_N_4_ could be investigated at 13.02° and 27.56° which were attributed to (100) and (002) crystal planes. While those at 22.70°, 32.36°, 40.04°, 47.44°, 54.14°, 58.22°, 67.46° and 78.4° were assigned to (101), (110), (021), (202), (211), (122), (220) and (312) lattice planes of LaNiO_3_. As revealed in Figure 1b, the diffraction peaks in Figure 1a can also be investigated in the patterns of Figure 1b. All the peak positions were well matched and no additional peaks appeared, which confirms the successful preparation of 30% LaNiO_3_/g-C_3_N_4_ (LG) and ALG photocatalysts. The average crystalline sizes of all the samples could be calculated by the Scherrer equation [25], and the detailed data could be found in Table 1. The average crystalline sizes of AgBr and LaNiO_3_ were 43.49 nm and 12.03 nm. The average crystalline size of x-ALG samples increase gradually from 20.56 to 26.47 nm because of the introduction of AgBr nanoparticles. 

**Figure 2:**
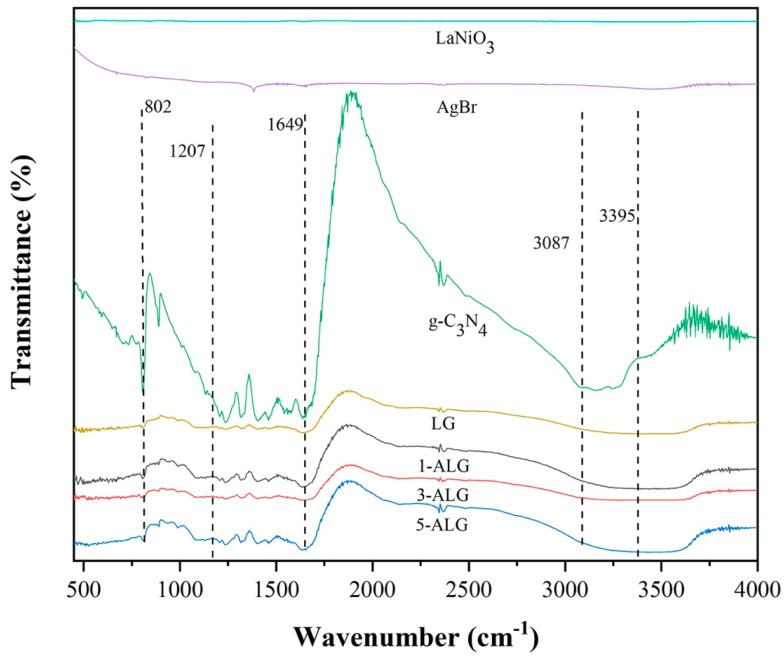
FT-IR spectra of bare AgBr, g-C_3_N_4_, 1-ALG, 3-ALG and 5-ALG.

The vibrational features of functional groups existing in the samples were analyzed by FT-IR. The FT-IR spectra of AgBr, LG and ALG were depicted in Figure 2, consistent with g-C_3_N_4_, the sharp band discerned at 802 cm^−1^ represented the triazine units breathing mode [15]. The absorption bands ranging from 1207–1649 cm^−1^ corresponds to the vibration of C-N stretching modes [30]. The peaks are caused by the stretching vibrations of heptazine-derived repeating units. The wide band ranging from 3087–3395 cm^−1^ could be attributed to N-H vibration modes [31]. 

Because AgBr and LaNiO_3_ were both inorganic materials and the stretching vibrations of them were hard to investigate by FT-IR. Based on this situation, TEM was employed to investigate the deposition of AgBr and LaNiO_3_ on the surface of g-C_3_N_4_. The characteristic bands of absorption water was overlapped by the absorption bands of g-C_3_N_4_, and the existent of water could confirmed by XPS [32].

**Figure 3:**
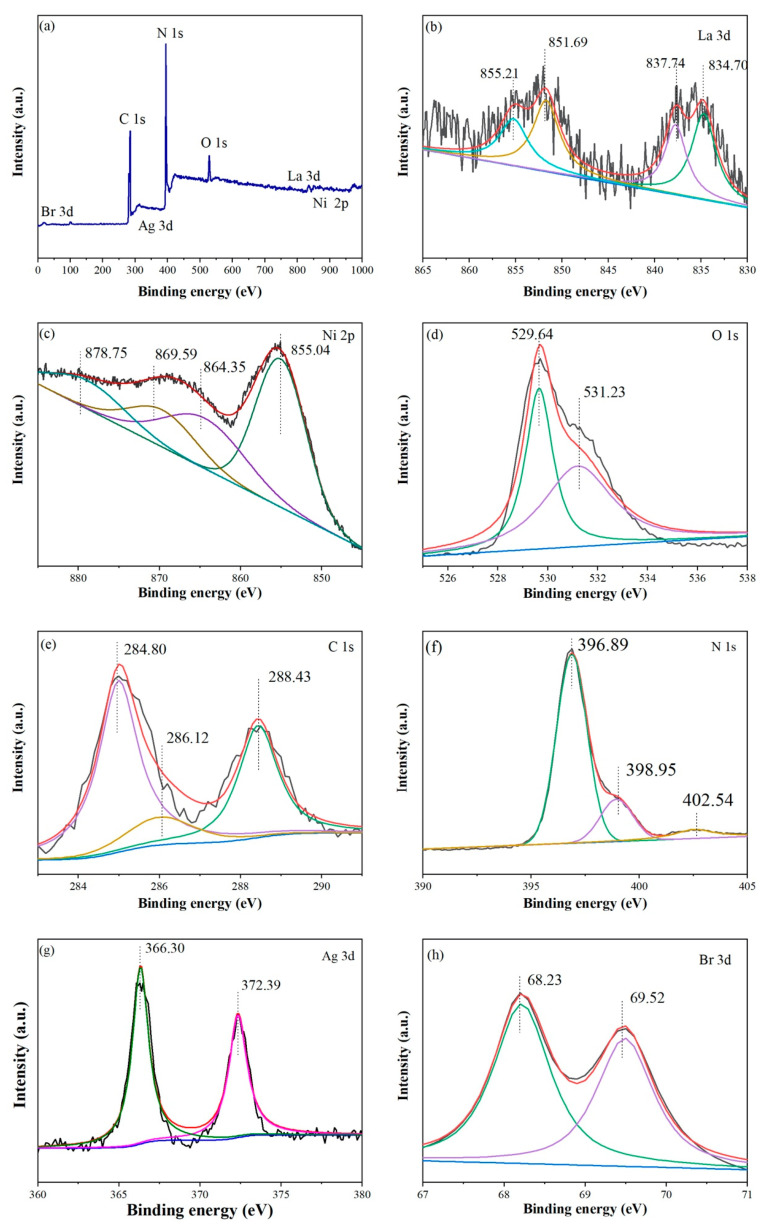
XPS spectra of as-obtained 3-ALG (**a**), La 3d (**b**), Ni 2p (**c**), O 1s (**d**), C 1s (**e**), N 1s (**f**), Ag 3d (**g**), and Br 3d (**h**).

The elemental composition of 3-ALG was examined by XPS and displayed in Figure 3a including La, Ni, O, Ag, Br, C, and N, which agrees with the chemical constituents of ALG. In Figure 3b, two pairs of shoulder peaks at 837.74, 834.70 eV and 851.69, 855.21 eV represent La 3d_5/2_ and La 3d_3/2_ [17]. The peaks at 855.04 and 869.59 eV are assigned to Ni 2p_3/2_ and Ni 2p_1/2_, also the satellite peak appeared at 864.35 and 878.75 eV, as shown in Figure 3c, indicating that Ni valence is +3 [33]. As shown in Figure 3d, The peaks at 529.64 and 531.23 are attributed to lattice oxygen and the chemisorbed oxygen from absorption water [33]. In Figure 3e, the peaks at 284.80, 286.12, and 288.43 eV are attributed to C-C coordination, sp^2^ hybridized carbon, and sp^3^ N-C=N group in g-C_3_N_4_. The N 1s spectrum is shown in Figure 3f, three peaks are fitted at 396.89, 398.95, and 402.54 eV, which are assigned to C-N=C, N-(C)_3_ and N-H groups, respectively [12]. In Figure 3g, two peaks could be observed at 366.90 and 372.39 eV, which were assigned to Ag 3d_5/2_ and Ag 3d_3/2_ [23]_._ Br 3d spectrum in Figure 3h displayed two peaks at 68.23 and 69.52 eV which represent Br 3d_5/2_ and Br 3d_3/2_ [22]. The results investigated by XPS could confirm the successful synthesis of 3-ALG sample.

**Figure 4:**
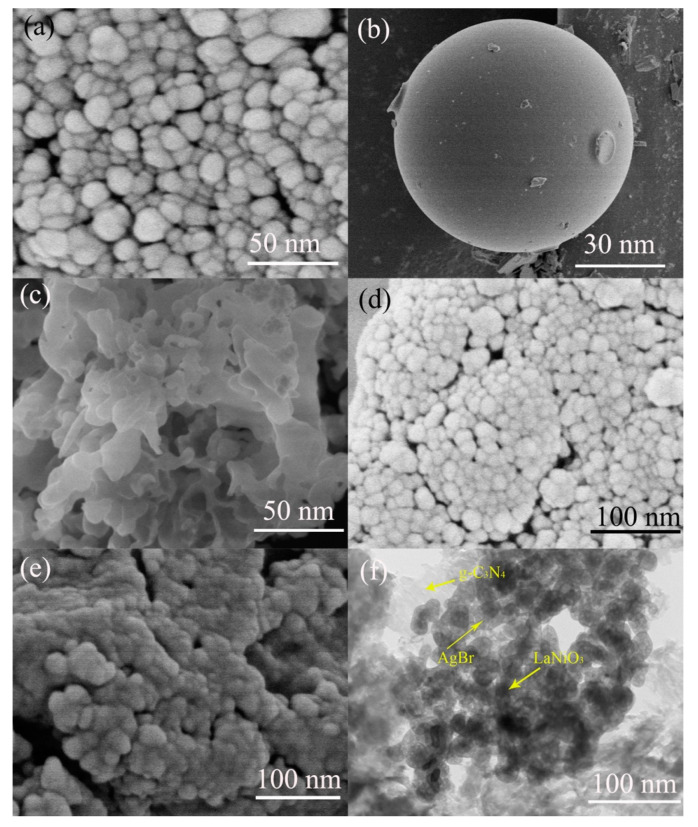
The SEM images of (**a**) AgBr, (**b**) LaNiO_3_, (**c**) g-C_3_N_4_, (**d**) LG, (**e**) 3-ALG and TEM image of 3-ALG (**f**).

To examine the surface microstructure and morphology, the morphological observation of AgBr, LaNiO_3,_ g-C_3_N_4_, LG and 3-AlG. The SEM and TEM images of samples were shown in Figure 4. In Figure 4a, AgBr displayed irregular-shaped nanoparticle structures with dimensions from 30–50 nm, which is consistent with the previously reported results. As examined in Figure 4b, LaNiO_3_ exhibited nanosphere structures with a smooth surface and a diameter of about 60 nm. As observed in Figure 4c, g-C_3_N_4_ showed the aggregated and slightly transparent layered nanosheet structures, which is the typical surface morphology of g-C_3_N_4_ nanosheets. As displayed in Figure 4d, LaNiO_3_ nanospheres were assembled with g-C_3_N_4_ indicating the formation of LG. After the addition of LaNiO_3_ and AgBr (Figure 4e), we found that LaNiO_3_ and AgBr were deposited on g-C_3_N_4_ nanosheets. The microstructure of 3-ALG was further characterized by TEM. The TEM result was shown in Figure 4f, in which darker LaNiO_3_ and AgBr were evenly grown on the surface of g-C_3_N_4_ demonstrating the successful formation of ALG ternary component hybrid. 

**Figure 5:**
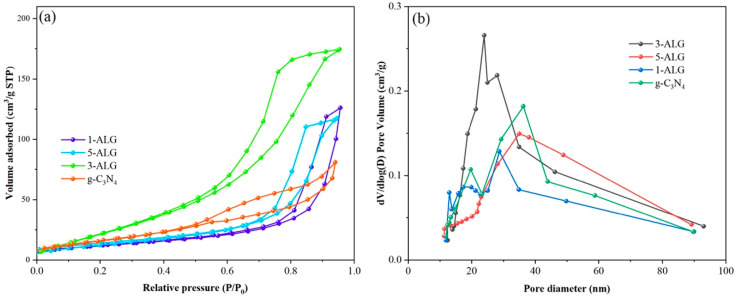
Nitrogen adsorption-desorption isotherms (**a**) and pore distribution curves (**b**) of bare g-C_3_N_4_ and 1-ALG, 3-ALG, and 5-ALG.

In order to examine the surface physico-chemical properties, the N_2_ adsorption-desorption isotherms and corresponding pore size distribution were illustrated in Figure 5. As shown in Figure 5a, according to IUPAC classification, all the samples demonstrated type-IV isotherm with H_3_ hysteresis loops, suggesting that all the samples presented typical mesoporous structure [33]. Among them, 3-ALG exhibited the highest adsorption isotherm, which demonstrates that sample 3-ALG possesses large pore size and tight intermolecular interactions of adsorbate molecules. The mesoporous structure of ALG composites and g-C_3_N_4_ was further proved by the plot of the pore-diameter distribution (Figure 5b) in which the average pore sizes of ALG composites and g-C_3_N_4_ were 27.99 nm (1-ALG), 27.50 nm (3-ALG), 26.19 nm (5-ALG) and 30.54 nm (g-C_3_N_4_) calculated by BJH model. The BET specific surface areas of g-C_3_N_4_ was shown in Table 1, which was calculated to be 27.32 m^2^/g. After coupling with AgBr and LaNiO_3,_ the BET specific surface areas increased from 45.81 to 65.39 m^2^/g and decreased to 30.72 m^2^/g. Because AgBr and LaNiO_3_ were assembled on the g-C_3_N_4_ nanosheets by ultra-sonic treatment, more microvoids were appearing on the surface of g-C_3_N_4_ nanosheets, the BET specific areas increased first. When the superfluous LaNiO_3_ was loaded on the surface of g-C_3_N_4_ nanosheets, partial pores of the hybrid were blocked leading to the decrease in the BET specific surface area. Generally, larger pore diameter and specific surface area are beneficial to the photocatalytic activity, which can adsorb more organics and increase the active sites for photocatalytic reaction.

### 2.2. Optical Properties

**Figure 6:**
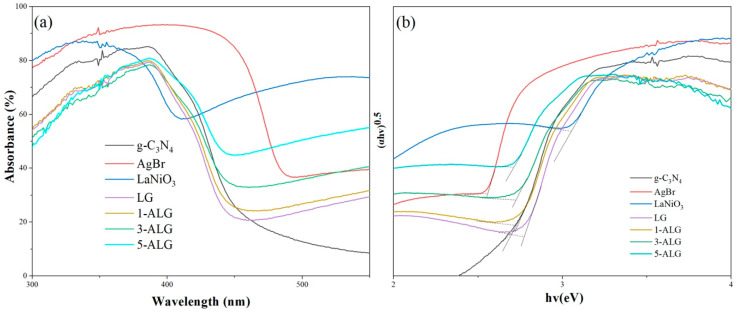
DRS spectra (**a**,**b**) the estimated band gap curves of all the samples obtained.

The UV-Vis DRS was employed to analyze the light absorption capacities of obtained samples. The results were illustrated in Figure 6a. What could be observed is that all the samples examined could be excited by both visible light and UV light. It could also be seen that the absorption edges of LaNiO_3_, g-C_3_N_4,_ and AgBr were about 441, 488, and 500 nm. The sample LG showed stronger absorption than bare g-C_3_N_4_. The optical absorption edge of ALG exhibited redshift which gradually became larger with the increasing amount of AgBr. The band gaps of all the samples were estimated by the Tauc/David-Mott model [34]. The band gap value of bare g-C_3_N_4_ is 2.73 eV, after coupled with LaNiO_3_ the value increased to 2.77 eV, furthermore, the band gap value became more smaller after the formation of ALG composites, the value of 5-ALG decreased to 2.70 eV even. Strong optical absorption capacity and narrow band gap are a benefit to the photocatalysis, so ALG hybrid will possess more enhanced photocatalytic performance than bare g-C_3_N_4_. 

Meanwhile, the band edge positions of CB and VB of all the semiconductor obtained could be calculated by Tauc equation [15]. The calculated CB and VB were shown in the following Table 2.

Distinctly, the valence and conduction band potentials of LaNiO_3_, AgBr, and g-C_3_N_4_ can be well-matched, making it possible to form a ternary component dual Z-scheme heterojunction composite photocatalyst, and the well-designed construction can suppress the recombination of photogenerated electrons and holes, promote the migration and transfer of photogenerated charge pairs, thereby enhancing the photocatalytic behavior.

**Figure 7:**
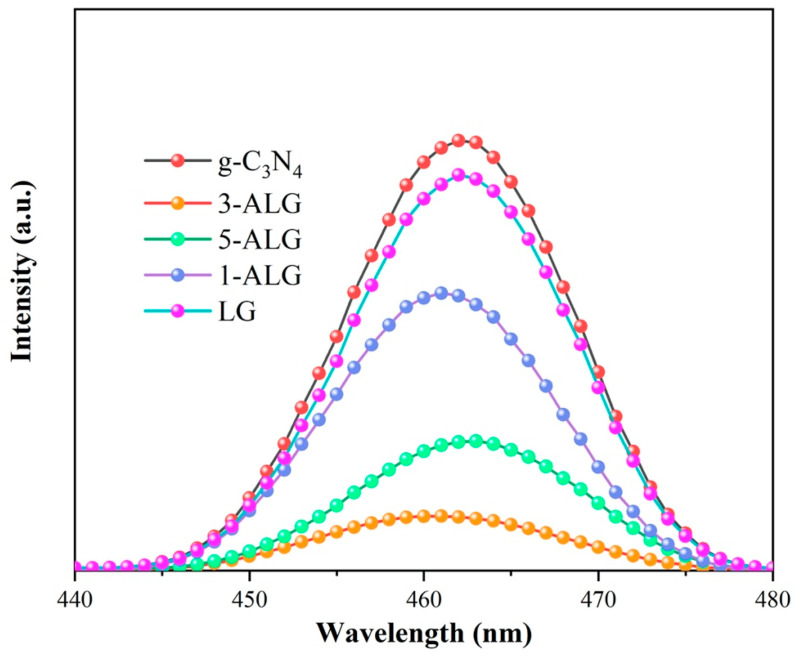
PL spectra of g-C_3_N_4_, LG, and 3-ALG.

The photoluminescence (PL) spectra were employed to investigate the photogenerated charge separation and migration in samples obtained by us with the excitation laser wavelength of 446 nm and the results were displayed in Figure 7. Generally, lower PL emission intensity represents lower recombination efficiency of photogenerated electrons and holes. As shown in Figure 7, the main emission peaks of g-C_3_N_4_ were located at around 460 nm, which was mentioned in the previous literature [15]. When compared with LG, 1-ALG, 3-ALG and 5-ALG, the PL peak intensity of g-C_3_N_4_ was stronger than them, and 3-ALG possessed the weakest PL intensity, which evidences that benefiting from the addition of LaNiO_3_ and AgBr, LG and 3-ALG possessed better separation and migration efficiency of photogenerated electrons and holes than bare g-C_3_N_4_. Furthermore, the PL spectra explained the best photocatalytic performance of 3-ALG in all the samples obtained. 

**Figure 8:**
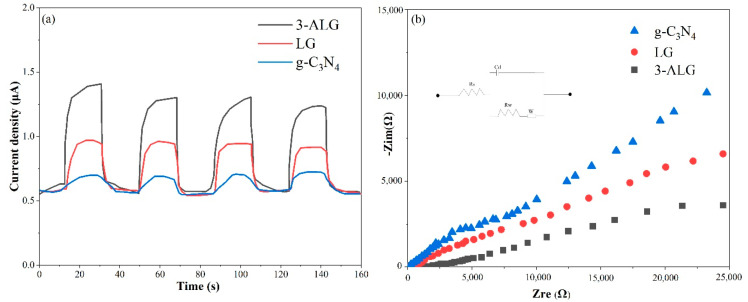
Photocurrent time curves (**a**) of g-C_3_N_4_, LG, and 3-ALG and EIS Nyquist plots (**b**).

The photocurrent response experiments of bare g-C_3_N_4_, LG and 3-ALG were employed to investigate the separation efficiency of photogenerated electrons and holes within the photocatalysts. As shown in Figure 8a, the transient photocurrent intensity of 3-ALG was much stronger than bare g-C_3_N_4_ and LG, which revealed that 3-ALG possessed the optimum separation efficiency for photo-aroused charge carriers. The migration and separation ability of photo-aroused electrons and holes within the samples was further investigated by the electrochemical impedance spectroscopy (EIS). As illustrated in Figure 8b, it is apparent that the arc radius on the Nyquist plot of sample 3-ALG is distinctly smaller when compared with bare g-C_3_N_4_ and LG, indicating the lowest transfer resistance and highest separation efficiency of charge pairs.

In short, considering the results of PL, photocurrent, and EIS, we found that by the construction of the ALG ternary component heterojunction, the effective separation of photogenerated electrons and holes were achieved, which can provide more photoactive electrons and holes for subsequent photocatalytic reactions. The phenomena have a positive effect on enhancing the photocatalytic performance of the photocatalyst. The conclusion can be verified in subsequent degradation experiments.

### 2.3. Photocatalytic Activity

**Figure 9:**
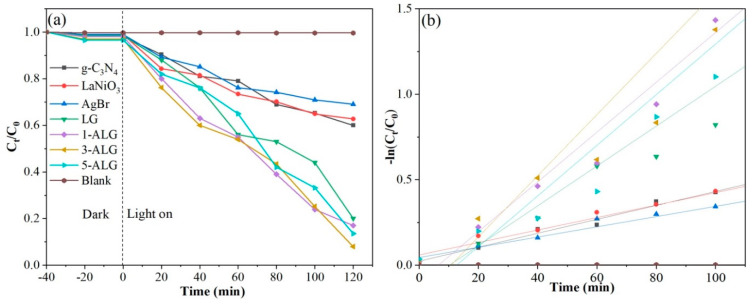
Photocatalytic performances (**a**) and the corresponding kinetic curves analyzed by the pseudo-first-order kinetic model (**b**) for NOR degradation over different photocatalysts.

Norfloxacin (NOR) is a typical antibiotic, which is wide-used, colorless, odorless non-biodegradable, and commonly detected in wastewater. In order to compare the photocatalytic abilities of all the photocatalysts, the photocatalytic degradation experiment of NOR was implemented under visible light. After 40 min the adsorption-desorption equilibrium away from light was gained, and the curves of NOR degradation over all the photocatalysts were recorded and demonstrated in Figure 9a. As displayed in Figure 9a, there is almost no degradation without any photocatalysts in suspension. When with bare g-C_3_N_4,_ AgBr, and LaNiO_3_, the degradation rates were still scant, reaching 40%, 38%, and 31%, respectively. While g-C_3_N_4_ coupled with LaNiO_3_, the photodegradation efficiency increased significantly, 80% of NOR was degraded within 120 min. The photocatalytic performance was enhanced further over the ternary component composite, the photocatalytic activities over ALG was 83% (1-ALG), 92% (3-ALG), and 87% (5-ALG). Obviously, the ternary component photocatalyst 3-ALG possesses the highest degradation ability towards NOR. The NOR photodegradation process was fitted by the pseudo-first-order kinetics. As displayed in Figure 9b and Table 3, the nice linear relationship between *−ln* (*C_t_/C_0_*) and *t* was gained using the model significantly. The apparent reaction rate constant of bare g-C_3_N_4_, AgBr, and LaNiO_3_ were merely 0.00408, 0.00365 and 0.00299 min^−1^, respectively. The degradation rate of NOR over LG hybrid was 0.01028 min^−1^ which is obviously faster than them. It is worth noting that all the ternary composite ALG showed the superior *k* value in which 3-ALG possessed the highest degradation rate of NOR up to 0.01790 min^−1^. It is obvious that a significant improvement in catalytic performance can be achieved due to the addition of AgBr and LaNiO_3_ on the surface of g-C_3_N_4_, which is benefited from the synergistic effect in the ternary heterojunction. The synergistic effect can inhibit the recombination of photogenerated holes and electrons, and promote the migration of photogenerated charge pairs. The photodegradation of NOR under visible light irradiation prove that the photocatalytic ability of x-ALG were higher than bare g-C_3_N_4_, AgBr, LaNiO_3_ as well as LG samples.

**Figure 10:**
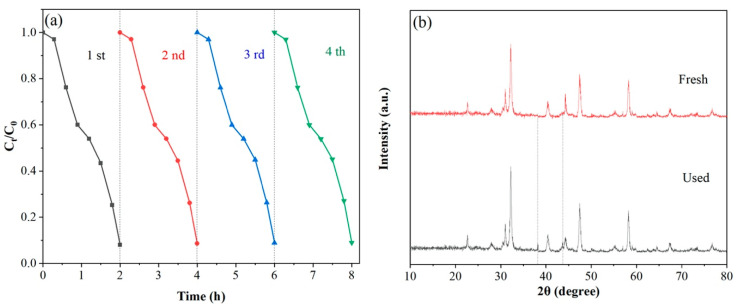
Recycling experiments (**a**) of the photocatalytic degradation of NOR over 3-ALG and (**b**) patterns of 3-ALG before and after sequential photocatalytic reaction under visible irradiation.

In the actual application process, the photostability and reproducibility are essential for the photocatalyst. The recyclability study on 3-ALG was implemented, and the results were depicted in Figure 10a. 3-ALG was reused for four times, for each recycling experiment, 3-ALG was centrifuged from the suspension and washed with ethanol and distilled water for several times, and dried before the next recycle. As demonstrated in Figure 10b, it is not difficult that after four times recycle experiments, the catalytic performance of 3-ALG did not decline significantly, and 90% of NOR can still be degraded within two hours indicating the stability of 3-ALG. It can be confirmed by the XRD patterns of 3-ALG before and after the experiments. We found that there is no obvious contradiction can be detected, which further illustrated the reliability stability of 3-ALG in practical applications. In addition, we found two peaks at 38.21° and 43.6° which can be associated with (111) and (200) crystal plane of Ag (JCPDS 040783) [35], which could be attributed to the reduction of Ag^+^ to metal Ag during the photocatalytic process. The metal Ag can act as a transit center for photogenerated electrons, which is advantageous for the photocatalytic reaction.

### 2.4. Photocatalytic Degradation of NOF Comparing with Other Materials

As an objective evaluation of our work, we compared the photocatalytic degradation of NOF over 3-ALG with other materials. According to the previous researches [4,36,37,38,39], FeVO_4_/Fe_2_TiO_5,_ CeO_2_/g-C_3_N_4_, CoWO_4_/g-C_3_N_4_, ZnS and BiWO_4_/WO_3_ were also taken and mixed with NOF aqueous solution under a 500 W xenon lamp equipped with a 420 nm cut filter for the degradation of NOF, which is as same as the degradation experiment over 3-ALG. As displayed in Table 4, apparently, the photocatalytic degradation of NOF over the materials demonstrated that the photocatalytic ability of 3-ALG under visible light illumination was at higher level.

### 2.5. Photocatalytic Mechanism

**Figure 11:**
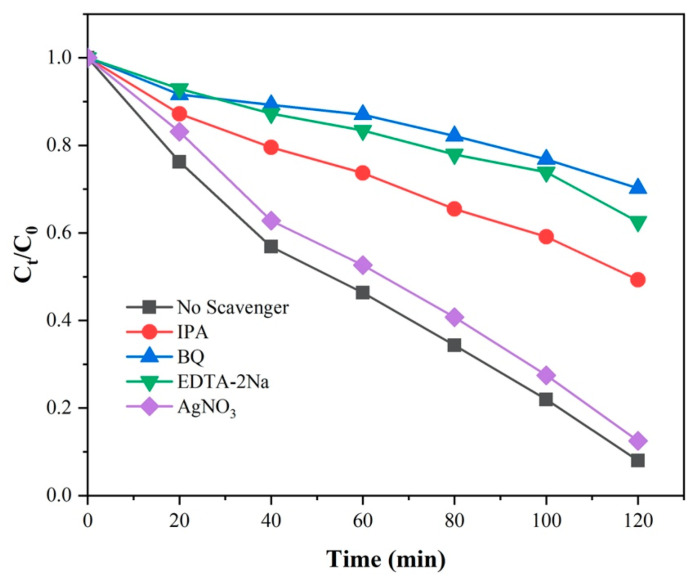
Radical quenching experiments over 3-ALG.

In order to analyze the significantly reinforced photocatalytic performance for NOR degradation over photocatalyst 3-ALG, the radical quenching experiments were conducted. Benzoquinone (BQ) as the scavenger for O_2_**^−^**, AgNO_3_ for e^−^, ethylenediaminetetraacetic acid disodium salt (EDTA-2Na) for h^+^, and isopropanol (IPA) for ·OH, were added to 3-ALG reaction system respectively. It is noteworthy that, although IPA can also react with h^+^ and O_2_^−^, according to many previous literatures [40,41], the main trapping radical of IPA in the radical quenching experiment is OH. The experiment results were illustrated in Figure 11, the photocatalytic degradation efficiency of NOR was noticeably reduced when BQ, EDTA-2Na and IPA involved in the reaction system. There were only small changes in the reaction upon the introduction of AgNO_3_, which was because the activity of photogenerated electrons in the reaction is limited by addition of AgNO_3_, some of electrons in the CB were also limited to produce O_2_^−^, the same phenomenon appeared in the photocatalytic reaction system of BiFeO_3_/ZrO_2_ [42]. From the above phenomena, O_2_^−^, h^+^ and OH can be identified as the main active species during the photodegradation process of NOR. Thus, the enhanced photocatalytic performance for NOR over ternary component dual Z-scheme heterojunction photocatalyst can be interpreted in terms of migration of photo-aroused charge pairs, the generation of h^+^ and the corresponding radicals in aqueous solution.

**Figure 12:**
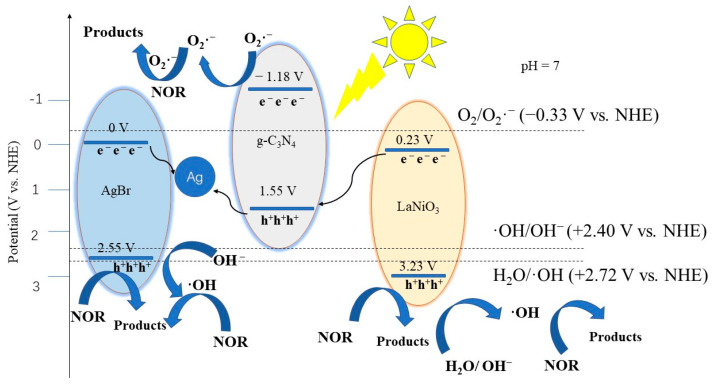
Proposed mechanism of as-prepared 3-ALG for the photocatalytic degradation of NOR under visible light irradiation (pH = 7).

The powerful photocatalytic ability for NOR over 3-ALG under visible light was demonstrated by the photocatalytic experiments, and the main reactive species within the degradation system were clarified by the analysis of the radical quenching experiments. Therefore, a possible dual Z-scheme photocatalytic mechanism is proposed in Figure 12.

As shown in Figure 12, in the phototcatalytic degradation process, Ag^+^ in AgBr is reduced to metallic Ag, moreover, metallic Ag can act as the photocatalytic charge pair transfer center of the dual Z-scheme. The e^−^ generated in the CB of AgBr can recombine with h^+^ migrating from the VB of g-C_3_N_4_ on metallic Ag. Similarly, the e^−^ in the CB of LaNiO_3_ can recombine with the h^+^ in the VB of g-C_3_N_4_. By this means, a large number of photo-generated electrons are accumulated in the CB of g-C_3_N_4_, while plenty of positive holes (h^+^) are retained in the VB of LaNiO_3_ and AgBr, which resulted in the efficacious separation of h^+^ and e^−^. Due to the CB potential of g-C_3_N_4_ is more negative than the potential O_2_/O_2_^−^ (−0.33 V vs. NHE at pH = 7), the e^−^ in the CB of g-C_3_N_4_ would reduce the O_2_ dissolved in water into O_2_^−^. Meanwhile, the h^+^ retained in the VB of LaNiO_3_ and AgBr could oxidize H_2_O and OH^−^ to produce OH, because the VB potential of AgBr was more positive than the reduction potential for OH/OH^−^ (+2.40 V vs. NHE) [12] and the VB potential of LaNiO_3_ was more positive than the reduction potential for OH/OH^−^ (+2.40 V vs. NHE) and H_2_O/OH (+2.72 V vs. NHE) [12]. The h^+^ can also be involved in degradation reactions due to its own strong oxidizability. As a result, O_2_^−^ and OH produced and h^+^ can decompose NOR into small molecules and eventually into CO_2_, H_2_O and NH_3_. The analysis above could be validated by radical quenching experiments. Consequently, it can be ascertained that 3-ALG ternary component photocatalyst adheres to a dual Z-scheme photogenerated charge transfer system. In this system, photogenerated holes and electrons are separated and migrated efficiently, while the photocatalyst retains a high oxidation-reduction capacity for contaminants.

## 3. Materials and Methods 

### 3.1. Synthesis of g-C_3_N_4_ Nanosheets

We synthesized g-C_3_N_4_ nanosheets based on the method described in the previous report [21]. Firstly, 10 g of melamine was placed in a covered crucible and heated at 550 °C for 4 h with a heating rate of 2 °C per minute in air. The bulk g-C_3_N_4_ achieved was ground into powder, subsequently put into a crucible without a lid and heated at 520 °C for another 2 h in air with a heating rate of 2 °C per minute to get g-C_3_N_4_ nanosheets. 

### 3.2. Synthesis of LaNiO_3_ Nanospheres

Typically, 1 mmol La(NO_3_)_3_·6H_2_O, 1 mmol Ni(NO_3_)_2_·6H_2_O and 5 mmol citric acid were dissolved in 200 mL deionized water with magnetic stirring for 2 h to achieve light green solution. The solution was poured into a 250 mol Teflon-lined stainless autoclave, heated at 180 °C for 12 h. The precipitate collected was washed with deionized water and ethanol for several times after cooling to room temperature and dried at 80 °C for 24 h and calcinated at 800 °C for 2h in air to achieve pure LaNiO_3_ nanospheres.

### 3.3. Synthesis of AgBr Nanoparticles

AgNO_3_ and KBr (1 mmol) were dissolved in 300 mL deionized water in dark with magnetic stirring for 30 min, washed by deionized water and ethanol for several times and dried at 60 °C for 12 h. AgBr nanoparticles were obtained.

### 3.4. Synthesis of LG Hybrid

LaNiO_3_ nanospheres (0.3 g) and 1 g-C_3_N_4_ nanosheets were mixed in 30 mL deionized water with magnetic stirring and ultrasonic vibration for 2 h. The resulting mixture was dried at 80 °C for 24 h to remove the remaining water, and calcinated in the air at 300 °C for 2 h. Then LaNiO_3_ nanospheres were successfully assembled on the surface of g-C_3_N_4_ nanosheets, the sample obtained was marked as LG. 

### 3.5. Synthesis of ALG Photocatalysts

Typically, 0.03 g AgBr, 0.3 g LaNiO_3_, and 1 g g-C_3_N_4_ was poured into 20 mL deionized water with magnetic stirring for 2 h, and endured for 12 h with ultrasonic vibration at room temperature. The mixture was dried at 80 °C for 24 h. After calcination in air for 3 h at 300 °C, we obtained ALG composites. By controlling the mass ratio of AgBr, the catalysts with a load of 1%, 3% and 5% of AgBr were accurately prepared and named as 1-ALG, 3-ALG and 5-AlG, respectively.

### 3.6. Characterization of Samples

We used XRD (Shimadzu, LabX-6000) to investigate the crystal structures of samples obtained. The chemical bonds on the surface of photocatalysts were examined by FTIR (Bruker, Vertex70, Karlsruhe, Germany). SEM (Hitachi, S-4800, Tokyo, Japan) and TEM (Tecnai, G2 F30, Hillsboro, OR, USA) were employed to detect the morphology of the samples. The element composition was characterized by XPS (VG-Multilab, 200, Madison, GA, USA). Nitrogen adsorption-desorption isotherms were conducted at 77 k using an ASAP 2460 surface area and porosity analyzer at liquid nitrogen temperature. We employed PL (Shimadzu, RF-6000, Kyoto, Japan) and UV-vis DRS (JASCO, UV-2600, Tokyo, Japan) to examine the photogenerated charge separation efficiency and optical absorption capacity. The transit photocurrent and electrochemical impedance spectroscopy (EIS) were conducted on an electrochemical workstation (Chenhua Instrument Corp, CHI 660E, Shanghai, China).

### 3.7. Photodegradation Measurements

The photocatalytic performance of as-obtained samples was tested by degradation experiments of NOR in aqueous solution. The visible-light source was provided by a 500 W xenon lamp equipped with a 420 nm cut filter. 

Typically, 20 mg of the sample as-obtained was added into 100 mL 20 mg/L NOR solution with magnetic stirring in a quartz tube reactor with a water circulation facility. Before light irradiation, the mixture suspension was remained in the dark for 30 min to reach the adsorption-desorption equilibrium between photocatalysts and degradation product. During the photodegradation process, 10 mL of the suspension was withdrawn and centrifuged to remove the photocatalyst each 20 min. The absorbance of the supernatant was examined at a wavelength of 280 nm. The photodegradation ratio for NOR was tested by the formula below:

*η* = (1 − *C_t_/C*_0_) × 100% (1)

where *η* is the photodegradation ratio for NOR, *C_t_* and *C*_0_ represent the initial and remaining NOR in aqueous solution, *t* is the light irradiation time, respectively. 

## 4. Conclusions

In conclusion, a novel ternary dual Z-scheme AgBr/LaNiO_3_/g-C_3_N_4_ photocatalyst was prepared via a facile ultrasound-assisted hydrothermal method. The introduction of AgBr promoted the optical absorption capacity of the heterojunction and made it possible for the metallic Ag to participate in degradation. Such a heterojunction possessed a high specific surface area of 65.39 m^2^/g, which can absorb more undecomposed molecules and provide photocatalytic reaction with more active sites. Metallic Ag involved in the reaction acting as the electron-holes transfer media to enhance the separation of charge pairs. A possible dual Z-scheme reaction mechanism was proposed, in which the light absorption and charge pairs separation were promoted. This novel ALG exhibited excellent photocatalytic performance for norfloxacin (NOR), 92% of NOR was degraded over 3-ALG within 120 min. In addition, ALG possessed remarkable stability and reusability. This study provided a feasible way to establish the ternary component dual Z-scheme g-C_3_N_4_-based photocatalyst and a possible method for photocatalysis in the decomposition of antibiotic-like contaminants in water.

## Figures and Tables

**Table 1:** The average pore sizes and specific surface areas of g-C_3_N_4_, 1-ALG, 3-ALG and 5-ALG.

Sample Name	Average Pore Sizes (nm)	Specific Surface Areas (m^2^/g)	Pore Volume (cm^3^/g)	Crystalline Size (nm)
g-C_3_N_4_	27.99	27.32	0.78	2.57
1-ALG	27.50	45.81	0.72	20.56
3-ALG	26.19	65.39	0.64	25.68
5-ALG	30.54	30.72	0.89	26.47

**Table 2:** The band edge positions of conduction and valence band for the sample g-C_3_N_4_, AgBr and LaNiO_3_ (pH = 7)_._

Sample Name	CB (V vs. NHE)	VB (V vs. NHE)
g-C_3_N_4_	−1.18	1.55
AgBr	0	2.55
LaNiO_3_	0.23	3.23

**Table 3:** Photocatalytic results and crystalline size of all the samples.

Sample Name	Degradation (%)	*k* (min^−1^)	Standard Deviation	Band Gap (eV)
g-C_3_N_4_	40	0.00408	0.01425	2.73
AgBr	31	0.00365	0.02134	2.55
LaNiO_3_	38	0.00299	0.02348	3.00
LG	80	0.01166	0.14129	2.77
1-ALG	83	0.01456	0.08799	2.73
3-ALG	92	0.01790	0.24918	2.72
5-ALG	87	0.01481	0.18515	2.70

**Table 4:** Photocatalytic degradation of 3-ALG, CeO_2_/g-C_3_N_4_, ZnS, FeVO_4_/FeTiO_5_, CoWO_4_/g-C_3_N_4_ and BiWO_4_/WO_3_.

Photocatalyst Name	Degradation Rate (%)	Photocatalyst Name	Degradation Rate (%)
3-ALG	92	FeVO_4_/Fe_2_TiO_5_	92
CeO_2_/g-C_3_N_4_	88.6	CoWO_4_/g-C_3_N_4_	97
ZnS	75	BiWO_4_/WO_3_	67
